# Multi-modality cardiac imaging confirms quadricuspid aortic valve and excludes papillary fibroelastoma

**DOI:** 10.1093/ehjimp/qyae025

**Published:** 2024-04-09

**Authors:** Malgorzata Wamil, Marcos Goncalves

**Affiliations:** Cardiac Imaging Department, Mayo Clinic Healthcare, 15 Portland Place, W1B 1PT, London, UK; Cardiology, Great Western Hospital NHS Trust, Marlborough Road, SN3 6BB, Swindon, UK; Cardiac Imaging Department, Mayo Clinic Healthcare, 15 Portland Place, W1B 1PT, London, UK

**Keywords:** multi-modality cardiac imaging, echocardiography, cardiac MRI, quadricuspid aortic valve, papillary fibroelastoma, transoesophageal echocardiography

A 26-year-old woman presented with a history of frequent sharp, left-sided chest pains, which were not associated with exercise. She had no significant past medical history. Her resting electrocardiogram (ECG) appeared normal, and the auscultation of her heart revealed no additional murmur. As part of her diagnostic workup, she underwent a transthoracic echocardiogram (TTE), which incidentally revealed a quadricuspid aortic valve (AV). It also raised suspicion of a mass attached to the cusps of the AV on the aortic side (*Figure 1A* and *B*) and suspected papillary fibroelastoma. To further investigate the anatomy of the AV, assess the ascending aorta, and characterize the suspected mass, cardiac magnetic resonance imaging (MRI) and a transoesophageal echocardiography (TOE) were performed. The TOE confirmed an X-shaped opening of the AV instead of the usual Y-shaped opening. The 3D TOE and CMR images clearly visualized the AV, showing a round opening. The valve was found to have four equal cusps, classifying it as a type A quadricuspid AV according to the Hurwitz and Roberts classification (*Figure 1C–G*). Doppler examination revealed a central jet of aortic regurgitation, which was quantified as mild based on various parameters (TTE colour Doppler and CMR phase contrast velocity AR regurgitation fraction 3%). Further imaging with CMR non-contrast aortogram confirmed no evidence of associated aortopathy (*Figure 1D, H*). Significantly, neither the TOE nor the CMR images identified any mass attached to the AV cusps (see Supplementary data online, *Video S1*). Instead, the thickening observed in the centre of cusp coaptation on the initial TTE images was confirmed to be consistent with the aberrant anatomy of the valve. This case highlights the valuable role of multi-modality cardiac imaging techniques in comprehensively assessing the anatomy and function of the AV. Considering those imaging techniques’ availability and cost, TTE will be used for annual follow-ups.

**Figure 1:**
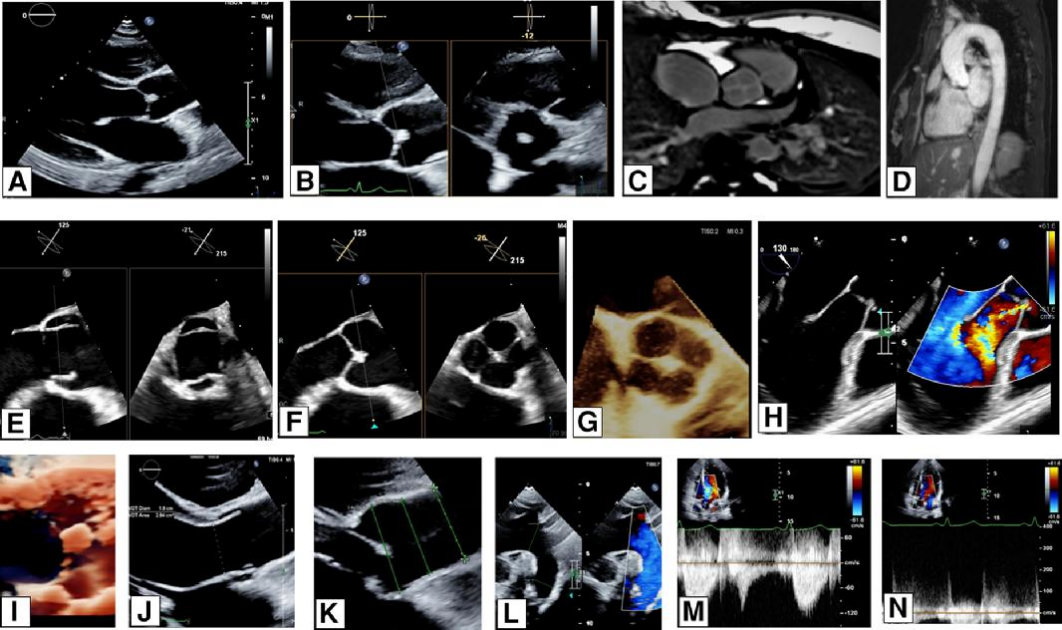
(*A*) Transthoracic echocardiography/parasternal long-axis view showing a suspicion of a papillary fibroelastoma attached to the AV. (*B*) Transthoracic echocardiography/parasternal long-axis with X-plane two-dimensional (2D) display view raising a suspicion of a papillary fibroelastoma attached to the AV. Note: unclear systolic AV morphology by 2D/colour flow mapping (CFM) Doppler assessment. Suspicion of quadricuspid AV in diastole. (*C*) Cardiac MRI anatomical images confirming the anatomy of the quadricuspid AV. (*D*) Cardiac non-contrast MRI aortogram showing normal size of the ascending aorta. (*E*) TOE X-plane 2D display: AV mid-oesophageal long-axis view showing a quadricuspid AV—systole. (*F*) TOE X-plane 2D display: AV mid-oesophageal long-axis view showing a quadricuspid AV—diastole, type A with no obvious evidence of papillary fibroelastoma. (*G*) TOE AV 3D zoom, showing AV quadricuspid morphology type A in diastole. (*H*) TOE AV mid-oesophageal long-axis view in colour compare mode showing mild AV regurgitation. (*I*) Transthoracic echocardiography/live 3D parasternal long-axis view showing a suspicion of a papillary fibroelastoma attached to the AV. (*J*) Transthoracic echocardiography/parasternal long-axis: 2D display view showing left ventricle outflow tract diameter—19 mm. (*K*) Transthoracic echocardiography/parasternal long-axis: 2D display view showing aortic root and proximal ascending aorta dimensions (sinuses of Valsalva 28 mm, ST junction 24 mm, proximal ascending aorta 25 mm, and distal aorta 23 mm). (*L*) Transthoracic echocardiography/suprasternal view: 2D display with colour Doppler compare showing aortic arch and proximal descending aorta dimensions with laminar flow present/no signs of aortic coarctation. Aortic arch diameter, 21 mm; proximal descending aorta diameter, 14 mm. (*M*) Transthoracic echocardiography/apical five-chamber view 2D display with continuous wave Doppler interrogation, showing normal transvalvular AV flow velocity. AV max velocity −1.2 m/s. (*N*) Transthoracic echocardiography/apical five-chamber view 2D display with continuous wave Doppler interrogation, showing no significant aortic regurgitation/suboptimal aortic regurgitation Doppler alignment. Transthoracic Echocardiography US Epiq CVx, Transducer X5-1 Transoesophageal Echocardiography US Epiq CVx, and Transducer X8-2t.

## Supplementary Material

qyae025_Supplementary_Data

## Data Availability

No new data were generated or analysed in support of this research.

## Supplementary data

**Consent:** The authors confirm that the patient has provided written consent for submitting and publishing this case report, including images and associated text, by the Committee on Publication Ethics (COPE) guidelines.

## Lead author biography

Dr Gosia Wamil is an Oxford-trained, internationally recognised consultant cardiologist and Assistant Professor of Medicine at Mayo Clinic. Her clinical specialities are heart failure and valvular pathologies. She is known for her cardiac MRI, CT and advanced echocardiography expertise and can often combine them in transformational ways to treat complex cardiovascular cases. Apart from her clinical career, Dr Wamil is also a clinician scientist with research experience spanning from molecular biology and drug development projects to large-scale clinical trials. She completed the British Heart Foundation (BHF) MSc-PhD program in cardiovascular science at the University of Edinburgh and a postdoctoral BHF Centre of Research Excellence fellowship in cardiac imaging at the University of Oxford. Her research interests include cardiovascular complications of diabetes and mechanisms of heart failure development in people with diabetes, using a variety of methodologies such as randomised controlled trials, AI and digital health technologies.

